# Factors affecting general practitioners’ referrals of patients to hip and knee arthroplasty: a focus group study from Northern Norway

**DOI:** 10.1186/s12913-025-12774-x

**Published:** 2025-05-20

**Authors:** Bodil H. Blix, Veronica Hovind, Tor Ingebrigtsen

**Affiliations:** 1 Department of Health and Care Sciences, Faculty of Health Sciences, UiT The Arctic University of Norway, Postboks 6050, Stakkevollan, Tromsø, 9037 Norway; 2 Helgelandssykehuset HF, Postboks 601, Mo i Rana, 8607 Norway; 3 UNN Tromsø, Postboks 100, Tromsø, 9038 Norway

**Keywords:** Free hospital choice, Shared decision-making, Patient leakage, Patient mobility

## Abstract

**Background:**

In Norway, patients have the right to choose their healthcare provider for elective specialized treatments, including the approximately 20,000 annual hip and knee arthroplasty surgeries. There is considerable regional variation in mobility – ‘patient leakage’ – out of a regional health authority for patients seeking these surgical procedures. General practitioners play a vital role in this decision-making process, acting as gatekeepers in referring patients to specialized healthcare. This study investigates the factors influencing Norwegian general practitioners’ referral decision for hip and knee arthroplasty surgeries.

**Methods:**

We conducted five focus group interviews with 28 general practitioners across various municipalities in Northern Norway, selected based on differing levels of ‘patient leakage’ according to the Norwegian Patient Registry. The thematic analysis focused on: 1. The involvement of general practitioners, patients, and other parties in the decision-making process, and 2. The impact of interpersonal, service-related, and broader societal factors on these decisions.

**Results:**

The analysis identified four main themes: 1. Navigating referral decisions, 2. Patients’ expectations, knowledge, and beliefs, 3. Service factors affecting referral practices, which includes sub-themes such as hospital reputation, general practitioners’ familiarity with specialists, selection bias, and concerns for post-operative care, and 4. Societal and structural factors, including geographic logistics and local hospital disputes.

**Conclusion:**

The decision-making process for selecting treatment providers is complex and influenced by multiple intersecting factors. While general practitioners are crucial in guiding patient referrals, many elements contributing to ‘patient leakage’ are beyond their control. Our results suggest that policies to address patient leakage should encompass a broader, cross-sectoral approach rather than focusing solely on healthcare services.

**Supplementary Information:**

The online version contains supplementary material available at 10.1186/s12913-025-12774-x.

## Background

In Norway, as in many other high-income countries, patients possess the right to select their healthcare provider for elective specialized treatments [1, 2], a policy known as ‘Free hospital choice’. The policy is expected to decrease waiting times, enhance quality and efficiency through strengthened competition, and foster equity in healthcare [3, 4]. General practitioners (GPs) play a crucial gatekeeping role in referring patients to specialized healthcare [5]. Shared decision-making is regarded as the ideal approach for making treatment and care choices. It is defined as “a collaborative process that involves the person using the service working with the healthcare professional to reach a joint decision about their care” [6]. Despite this, research indicates that few patients actively select their healthcare providers, and many do not view having a choice as important [3]. Additionally, studies have shown that patients often rely on their primary care provider to recommend high-quality specialized care [7, 8], and it is assumed that GPs are more informed about treatment options and quality of care than their patients [5, 9]. A recent systematic review revealed that when choosing a specialist for their patients, referring providers consider not only the specialists’ clinical expertise but also subjective factors. These include past interactions that both the patient and the referring physician have had with the specialist, which influence their assessment of the specialist’s quality [8]. A comprehensive survey that included 7,183 GPs across 31 European countries, as well as in Australia, Canada, and New Zealand, found that GPs’ referral decisions are most influenced by patient preferences and the GPs’ own previous experience with specialists. The use of benchmark information to inform referrals is rare, and in Norway, it is almost never used [10].

The Norwegian healthcare system is predominantly publicly funded via taxes through block grants and activity-based funding, each contributing about half of the total funds [2]. The public specialized healthcare services are managed as local enterprises within four regional health authorities. These authorities are responsible for the provision of specialized healthcare for the population in their respective regions, either directly or via contracts with private providers. If patients opt for treatment from a provider outside their local enterprise or health authority, the funding designated for their care follows them to the chosen provider. Furthermore, the national insurance scheme reimburses patients for travel expenses related to their treatment. The right to choose treatment provider extends to the approximately 20,000 patients who annually require hip (11,000) and knee (9,000) arthroplasty surgeries [11]. The Norwegian Health Atlas has highlighted considerable regional variation in mobility, often referred to as ‘patient leakage’ [12] out of a regional health authority, for patients seeking these surgical procedures [13]. The choice of treatment provider is aided by information on predicted wait times available on a public website [14]. The published wait times for arthroplasty procedures vary significantly, ranging from four weeks to over a year, with no obvious pattern of differences across health regions. Arthroplasties are standardized surgical procedures, and annual reports from the Norwegian Arthroplasty Register indicate minimal variation in practice between hospitals and health regions [11].

Although there is substantial evidence documenting the prevalence of and regional variations in ‘patient leakage’, there is limited understanding of the decision-making processes that guide choice of treatment provider. In this study, we specifically explore the factors affecting Norwegian GPs’ decision-making processes regarding where to refer patients for hip and knee arthroplasty surgeries, with a particular attention to referrals to treatment providers located outside their local regional health enterprises.

## Methods

### Study context

This study was conducted in Northern Norway, an area under the jurisdiction of Northern Norway Regional Health Authority. Less than 500,000 people, or about 10% of Norway’s population, reside in Northern Norway [15]. This region spans roughly 35% of the country’s geographical area and is characterized by widely dispersed settlements and significant distances between communities and local public hospitals. Transportation options within the region and between the region and the southern part of the country vary, with most residents relying on plane, boat, car or bus.

Public specialist healthcare services in Northern Norway are managed by Northern Norway Regional Health Authority through four hospital enterprises. There are two to three local hospitals within each enterprise, and a varying degree of centralization of orthopedic surgery including arthroplasties within each enterprise:

- Finnmark hospital enterprise, with somatic hospitals in Hammerfest and Kirkenes, and decentralized specialist healthcare in Alta.
- The University Hospital of North Norway, with a tertiary regional referral center in Tromsø and local somatic hospitals in Narvik and Harstad.
- Nordland hospital enterprise, with somatic hospitals in Bodø, Gravdal and Stokmarknes.
- Helgeland hospital enterprise, with somatic hospitals in Mo i Rana, Mosjøen and Sandnessjøen.

This article is based on focus group (FG) discussions with GPs from five municipalities in Northern Norway. It contributes to a broader research initiative investigating patient mobility, also known as ‘patient leakage’, specifically among patients requiring hip or knee arthroplasty. Alongside FG discussions with GPs, the research initiative incorporates data from The Norwegian Arthroplasty Register, The Norwegian Patient Registry, and Statistics Norway, and individual qualitative interviews with patients and orthopedic surgeons.

### Participants and recruitment

We enlisted a patient research partner via the user panel of the health enterprise where the second author works. This partner was invited to engage in all phases of the research process and contributed to the study design, including the guide for conducting the FG discussions.

The study included five municipalities; three located in Finnmark County and two in Nordland County. These municipalities were intentionally selected due to the differences in the levels of ‘patient leakage’ as recorded in the Norwegian Patient Registry. The municipalities included have populations ranging from 7,400 to 26,000 [16].

We aimed to recruit GPs with a range of work experience, and to achieve this, we enlisted the help of key municipal personnel, such as municipal chief medical officers and physicians responsible for the specialist training of GPs, to identify potential participants. The second author reached out to potential participants, providing them with written and oral detailed information about the study. Upon obtaining written consent from the participants, the second author scheduled the FG discussions.

The composition of the focus groups is presented in Table 1.

**Table 1:** Focus groups

Focus group	County	Number of participants
1	Finnmark	5
2	Finnmark	7
3	Finnmark	5
4	Nordland	6
5	Nordland	5

In some cases, the FGs were composed of GPs from various offices or healthcare centers within the same municipality. In contrast, in smaller municipalities, the FGs included most GPs working there.

### Focus group discussions

To moderate the discussions, a comprehensive topic guide was employed, covering several areas:

1. The GPs’ referral practices: This included their preferences for treatment providers, sources of information and advice on choosing providers, considerations of travel distance and costs, and decision-making processes.
2. Shared decision-making: Topics involved evaluating patients’ understanding of their illness and treatment options, involving patients in the decision-making process, assumptions about the significance of the treatment location for patients, how GPs facilitate patients’ choice of provider, and circumstances in which disclosing the right to choose a provider might be deemed inappropriate.
3. Collaboration with hospitals: Discussion points included the nature of the GPs’ collaboration with local hospitals, their thoughts on what makes a hospital an attractive option for treatment, and ideas for enhancing the partnerships between hospitals and primary healthcare services.
4. Societal factors: The guide also prompted discussions on how societal issues, such as political processes and discussions regarding the localization of local hospitals, influence GPs’ referral practices.

The content of the topic guide was developed based on the overarching research initiative’s focus on ‘patient leakage’. While registry data can provide answers on whether ‘patient leakage’ is occurring, and potentially between which regions and hospitals, a qualitative study can offer insights into some causes of this phenomenon. Based on the central role of GPs in referring patients to specialist healthcare services, we assumed that their practices could both promote and prevent ‘patient leakage’. We assumed that GPs’ referral practices were based on professional and clinical assessments, but we were also curious about whether and how these practices were shaped by factors outside the GP’s office, such as policies on shared decision-making, and GPs’ and patients’ experiences with and assumptions about local hospitals and other specialist healthcare services.

The FG discussions were facilitated by the second author, who acted as the moderator, with a research assistant who served as co-moderator. These discussions took place in meeting rooms within the local healthcare centers. While the second author guided the conversation, the research assistant concentrated on observing the participants’ interaction and documenting the proceedings. Following Barbour [17], we believe that a crucial aspect of FGs is that the interaction should primarily occur among participants rather than between participants and the researcher(s). All participants were active and engaged in the FG discussions, although some were more active than others. They encouraged and challenged each other’s perceptions and statements through both verbal and non-verbal expressions.

All FG sessions were recorded for accuracy and further analysis.

### Analysis

The following research questions guided our analysis:

1. How do GPs describe their involvement, their patients’ participation, and the contributions of other relevant parties in decision-making processes for referring patients to arthroplasty surgery?
2. How do interpersonal, service, and broader societal factors shape these processes?

While the first, broader research question guided the development of the topic guide, the second research question emerged during the focus FG discussions and the initial phases of analysis. The audio recordings were transcribed verbatim. The lead author repeatedly read through the transcripts to become thoroughly acquainted with the content. Following the approach outlined by Braun and Clarke [18, 19], thematic analysis was deemed appropriate to engage with the data. The lead author individually assessed each transcript from the focus group discussions, identifying and coding text segments pertinent to the research questions. This process of identifying patterns and contradictions within the data involved a meticulous and iterative review of both the full data set and the coded segments. Subsequently, the lead author grouped the codes into preliminary themes. These initial themes, along with the related data excerpts, were then shared with the author group for feedback and further discussion. The next step was a collaborative effort where all authors worked together to refine, clarify, and name the themes. This cyclical approach fostered a dynamic interaction with the data, allowing for both in-depth analysis and critical reflection.

While thematic analysis typically centers on the content of *what* participants say, it is important to recognize that focus groups also leverage group dynamics – *how* people discuss specific topics [20, 21]. Focus groups “afford researchers access to social-interactional dynamics that produce particular memories, positions, ideologies, practices, and desires among specific groups of people” ([21]: 559). With this understanding, we have carefully examined the group dynamics in our analysis. The notes from the FG discussions offered cues for passages that required particular attention. Additionally, in presenting our findings, we have endeavored to highlight these dynamics by including not only individual statements but also the conversational context in which they occur.

The diverse expertise of the author group – comprising the lead author’s background in health services research and expertise in qualitative methods, the second author’s specialization in orthopedics, and the third author’s experience with leadership in healthcare – proved to be a significant advantage during the interpretation phase.

## Results

The GPs in our study navigated their referral practice for hip and knee surgery within the frames of the ideal of shared decision-making and the patients’ legal right to choose their hospital. However, our analysis revealed that the GPs’ referral practices were shaped by various intersecting factors at the macro, meso and micro levels. We identified four main themes with several sub-themes.

The themes and sub-themes are outlined in Table 2.

**Table 2:** Themes and sub-themes

Themes	1. Navigating referral decisions	2. Patients’ expectations, knowledge, and beliefs	3. Service factors affecting referral practices	4. Societal and structural factors affecting referral practices
Sub-themes			3.1 Hospital reputation	4.1 Geographic and logistic factors
			3.2 GPs’ familiarity and contact with specialists	4.2 Local hospital disputes
			3.3 Selection bias	
			3.4 Concerns for post-operative care	

### Navigating referral decisions

The GPs in our study recognized their pivotal role as gatekeepers in the referral process for hip and knee surgery. They felt well-positioned to advise patients, given their familiarity with the patients’ histories and life circumstances. One participant highlighted the significant influence GPs have, noting, “The GP undoubtedly has significant impact on where the patient travels. The information… When you’ve had the same GP for many years, you generally trust them. If not, you switch [to another GP]” (FG 5). This participant also affirmed that patients are consistently involved in the decision-making process: “Obviously! We always ask the patient if they have any specific preferences for their referral destination” (FG 5).

However, other parts of the FG discussions revealed that involving patients in the decision-making process about hospital choice is not as straightforward as suggested. Several GPs indicated that they seldom discuss the choice of hospital with their patients. One participant explained:

I rarely discuss with the patient… my patients where they will have surgery. I don’t see a need to question it. I have no reason to recommend another hospital than [the local hospital]. It rarely comes up. The entry point to hospital choice is that there is an indication for surgery. After that, people can do what they want. And some do, I know (FG 1).

Further discussions revealed that GPs seldom are the ones initiating conversations about hospital choice unless specific conditions prompt it. During one exchange, the GPs shared:

1: I never bring up the topic

2: I never do.

3: Perhaps I do, sometimes. It depends.

1: Perhaps, only if the waiting times are terribly long.

3: Yes, and especially if the patient is working. Younger people.

1: Yes.

(FG 2).

This dialogue suggests that GPs typically discuss hospital options only under exceptional circumstances, such as unusually long waiting times or when employment considerations are at stake, indicating that such discussions are more the exception than the norm. Additionally, the FG discussions revealed that GPs generally prefer referring patients to the nearest hospital, considering other options mainly when prompted by the patients themselves:

1: I think it’s great that the option to choose hospital exists. Free hospital choice. However, having local services is tremendously important. That’s actually the most important aspect. But Free hospital choice is a supplement to the local hospital.

2: I only briefly mention that if the patient complains about long waiting times. Then I say, ‘You can use Free hospital choice. That’s all I inform about, that they have options beyond the waiting time at the local hospital.

(FG 3)

Furthermore, it appears that GPs view themselves as supporters of local hospitals, only facilitating a change when patients express a clear desire to seek treatment elsewhere. One GP noted, “I believe it must be the patient who has a motivation to get out of the local system. I don’t stop them, but I don’t motivate them either” (FG2).

The FG discussions revealed that the GPs primarily viewed their responsibility as ensuring patients are referred for treatment. One GP expressed:

Hospital choice doesn’t engage me much, I must admit. It’s not something I actively think about. My main concern is to get patients to an orthopedist quickly, and fortunately, it usually doesn’t take long. I don’t consider other options unless the patients themselves suggest it (FG 3).

This comment suggests that GPs view the decision of which treatment provider to choose as peripheral to their responsibilities. Additionally, some GPs even questioned their duty to inform patients about their right to choose a hospital. This uncertainty is illustrated in the following exchange:

1: People know about their right to choose hospital nowadays!

2: Are we obligated to inform about this right before we refer?

1: I don’t.

2: I’m asking you… I don’t know.

3: There are some patients that are a lot of hassle.

2: I don’t do it consistently.

1: Neither do I.

(FG1)

This dialogue indicates inconsistency and uncertainty among GPs regarding their obligation to inform patients of their hospital choice rights. This stands in stark contrast to the initial statement from one of the participants who claimed that they always inquire about patients’ specific preferences for referral destinations.

### Patients’ expectations, knowledge, and beliefs

The first theme reveals that GPs are primarily focused on referring their patients for treatment and are less concerned with discussing which hospital to choose. Instead, the responsibility for initiating discussions about selecting a different hospital than the one initially chosen by the GP typically falls to the patients, and the FG discussions showed that patients do indeed initiate these discussions for various reasons.

GPs noted that many of their patients are well-informed and knowledgeable about health matters, including surgical techniques. For example, some patients express interest in specific surgical methods, often having researched these topics before their consultations. According to the GPs, this trend of informed patients is on the rise, with individuals understanding their medical options and making choices as if selecting from a menu when approaching the healthcare services:

1: Some patients are interested in specific surgical techniques being used.

Interviewer: Could you please elaborate on that?

1: For instance, some are specifically concerned whether the anterior approach is employed.

Interviewer: Do you think these patients have read about this before they come to you?

1: Yes, some definitely have.

2: I’ve noticed an increase in this trend. Patients are well-versed in the medical aspects. They are aware of the available options and often make choices as if selecting from a menu when they approach healthcare services.

(FG4)

Additionally, many patients are aware of waiting times and the experience levels of different hospitals. They actively seek out this information online, with some expressing a preference for hospitals known for high volumes of specific surgeries, such as hip replacements:

1: There is information available on the internet about where the largest volumes of surgeries are performed.

2: That’s something else.

1: It’s there. Many patients look at it. I have patients who have expressed a desire to be treated at [hospital in south-eastern Norway] based on such numbers. Clearly, we’re dealing with a well-informed population, especially among younger patients. For them, it’s important to go to places with a high caseload on hip prosthesis. There’s no doubt about that.

(FG5)

While some patients do actively seek information about hospitals’ caseloads, waiting times, and surgical techniques, some GPs observed that myths also play a role in patient preferences, such as the belief that healthcare services are superior in the southern parts of the country. This perception leads some patients to insist on being referred to hospitals in those regions: “People were obsessed with going to the south, down to where everything is better, you know. Everything is better in the south [ironic voice]” (FG1). Other GPs pointed out that some patients prefer to have surgery at hospitals other than their local ones due to a perceived inferiority complex. This sentiment is prevalent across hospitals of various sizes and locations:

1: [The local hospital] is obviously seen as a little brother to the university hospital. Many people hold this belief and think it’s better to have surgery elsewhere due to mistrust.

2: Right.

1: They think [the local hospital] just wants to cling to the patients.

2: Because of the money.

1: From what I’ve seen with the patients I meet, that seems to be the case. I noticed the same thing when I worked at the university hospital. People wanted to go to the south. It seems no matter where you are, there’s always somewhere else perceived as better.

3: Right. And I don’t believe people base these judgements on actual knowledge.

4: It’s more of a gut feeling.

3: A feeling that somewhere else is better.

(FG2)

According to the GPs, there has been a noticeable shift in patients’ awareness of their rights and their desire to be involved in decision-making processes, impacting GPs’ referral practices. Patients are now more likely to come to consultations with specific requests, having already decided on their preferred treatments and hospitals:

I believe patients are more… are more informed. That’s my impression. Previously, patients would come in and say, ‘I’ve waited so long. Isn’t there anything we could do?’ And I could suggest, ‘There’s always the option of Free hospital choice’. But now, it seems patients arrive having already made up their minds; ‘I’ve thought about travelling’.

(FG1)

This change reflects a broader trend in healthcare where patients are more assertive about their needs, as demonstrated in the following exchange:

1: I’m not sure about prosthesis, but it’s my impression that people are more demanding now. They come in with specific requests. Maybe not orders, but they’ve decided in advance, ‘I want this and that, I might discuss this, but I’m definitively having that’. Previously, it was more a question of, ‘Do you think this could be right for me? What do you think?’ Now, a larger share of the patients isn’t interested in others’ opinions because they already have made up their minds.

2: They’ve already decided before they even walk through the door that they want that x-ray.

1: Yes. ‘My knee hurts, so I must have an MRI’.

(FG5)

GPs also noted that their patients have specific expectations regarding the timing of surgeries to accommodate their personal schedules:

They want their surgeries to accommodate their busy schedules. They have their ‘migratory bird trips’ to Spain in the winter and don’t want anything to interfere with their summer boat trips and other activities. This is the modern retiree – they want it like this. Retirees are like this. And in the waiting room, no one is more eager to get moving than they are. Their schedules are packed, and they are focused on managing their own lives now (FG4).

Overall, these discussions indicate a recent trend in GP practices, influenced by patients’ increased knowledge assertiveness and specific healthcare demands across various age groups.

### Service factors affecting referral practices

While the previous theme highlighted how GPs’ referral practices are influenced by patients’ expectations and behaviors, discussions also touched on service-level factors affecting these practices. These include hospital reputation, the GPs’ familiarity with and contact with specialists, and concerns about post-operative care.

#### Hospital reputation

As noted earlier, GPs observed that some patients base their treatment preferences on their knowledge of hospital characteristics such as waiting time, caseload, and surgical techniques. However, the GPs also provided a more nuanced view of their patients’ requests, suggesting they are not solely based on hard facts:

1: I don’t think you should underestimate the fact that patients talk with each other at the seniors’ café.

2: [laughs] Yes.

1: Or at the senior gym, the senior dance, and other places. They have heard things like, ‘My neighbor or my friend had surgery there, and I want to go there too.’

(FG 5)

Patients often base their treatment requests on the experiences of others, and hospital reputations are influenced not just by treatment outcomes but also by patients’ overall hospital stay experiences: “I’ve received a lot of feedback about the negative tone in some hospital departments, where everything is a hassle and there isn’t a positive atmosphere among the care staff” (FG4).

According to the GPs, hospital reputations, whether positive or negative, sometimes take on a life of their own and persist over time, regardless of changes in reality:

Some patients outright refuse to go to [hospital name], saying, ‘They have a lot of infections there. It will only cause problems.’ Whether that’s true or not, I can’t say, but that’s their perception. And it sticks in people’s minds as a place of complications and infections. (FG5)

The GPs also noted that hospital reputations are affected by patients’ experiences in other parts of the healthcare system:

1: Todays retirees are very active. They share stories on Facebook and in their hiking groups, so one mishap can have significant repercussions.

2: It doesn’t even have to be a mishap.

1: Right. If rehabilitation progresses slowly, it might actually be due to poor follow-up from the physiotherapist, but the orthopedic surgeon gets blamed because he was the one who used the knife.

2: People love to talk about their health, about their experiences, so negative experiences spread quickly.

(FG4)

According to the GPs, the spread of hospital reputations is now faster, wider, and more sustained than in the past, partly due to social media: “With Facebook. You didn’t have the same kind of rapid spread before. I believe the impact is much greater now” (FG1), and some mentioned that “rumors spread like wildfire” (FG1). This suggests that hospital reputation is somewhat beyond the control of GPs and hospitals. However, the GPs were mindful not to contribute to the perpetuation of these reputations:

Of course, we have discussed among ourselves the importance of remaining neutral so as not to reinforce these perceptions. Obviously, our words have a tremendous impact in the population (FG4).

Some GPs also actively confront hospital reputations during patient consultations:

When someone says they don’t want to go to [hospital name], I ask ‘Why? Do you have any negative experiences?’ If they just say, ‘No, I’ve just heard a lot’, I usually respond, ‘They are skilled, and I have many patients who have been there, and the results are okay’ (FG2).

However, the GPs acknowledge that other healthcare professionals can contribute to the establishment and perpetration of hospital reputations:

Physiotherapists! We know some of them prefer specific centers in the south and talk to patients about it. This definitely influences the patients. There is no doubt that physiotherapists contribute to patients ending up in other health enterprises (FG5).

In summary, while the GPs saw themselves as actively referring patients to local hospitals, they portrayed patients’ requests for referrals to other hospitals as being more influenced by the hospitals’ reputations. Although they considered themselves as influential in guiding patients’ choices, they aimed to stay neutral. However, they recognized that hospital reputations were shaped by factors beyond their control, which were reinforced by both patients and other healthcare professionals.

#### GPs’ familiarity and contact with specialists

The GPs generally preferred referring patients to the local hospital, primarily due to its proximity and their familiarity with the specialists there. The following dialogue illustrates this preference:

1: I don’t advocate for Free hospital choice. I don’t say, ‘Remember, you can always choose to travel elsewhere’. I think, as long as the local services are professionally sound….

2: You promote the local hospital? So do I.

1: That’s how I feel. It’s here, and it’s close by.

2: And I know the orthopedic surgeons here and have a good dialogue with them if there are any complications.

3: Exactly! It’s more practical if something happens in the aftermath.

(FG2)

The GPs also communicate their familiarity with the local hospital and specialists to their patients, as shown in the following exchange:

1: When patients consider Free hospital choice, I tell them I’m not familiar with the practices at other hospitals.

2: I do the same. If people ask, ‘Where are they most competent on this?’, I usually say I don’t have an opinion on that.

3: And then I say, ‘but I do know that those particular surgeons in [local hospital] are very skilled. I know nothing about [hospitals in southern Norway], but I do know that they are very skilled at [local hospital].

(FG2)

This discussion highlights that GPs not only share their knowledge of local hospital professionals with their patients but also their lack of information about other healthcare options.

While the GPs emphasized the importance of knowing the local surgeons’ expertise, they also noted that the surgeons’ level of expertise was not their primary concern:

1: I’m aware that that the orthopedic surgeons who come here are well known and experienced within the system. They’ve been operating for a long time. But I wouldn’t call them trailblazers in the field. For instance, I’m not convinced they are the first to implement new prostheses. So….

2: No, but that’s not necessarily their role.

1: No.

(FG1)

More than specific surgical expertise, the GPs valued continuity and stability in the local surgical staff when referring patients to the hospital:

It’s obviously important to have a professional environment. It’s reassuring to know that the environment is robust and stabile, right? Seeing the same professionals’ names year after year and having ongoing dialogue with them creates a sense of safety. It does (FG5).

Consequently, extensive use of short-term locum tenens in the local hospitals made the GPs feel less secure:

1: When they are regulars, people we know, we trust them. But with locum tenens, we know little about where they come from and where… I don’t feel safe.

2: Mmmm….

3: There’s generally a good reason to be wary when someone only works temporarily in orthopedic surgery. For example, someone from Denmark who travels here for a week, then somewhere else the next. It raises questions.

1: However, I have no doubts about regular temporaries like [person name]. I wouldn’t hesitate with him. But he’s not a temporary anymore, he’s a regular now. Some locum tenens come back repeatedly, and you get to….

3: You get to know them.

(FG2)

Additionally, some GPs noted that familiarity with local orthopedic surgeons from informal and non-professional settings increased their likelihood of referring their patients to the local hospital:

1: I wonder if I would feel differently if I lived elsewhere, in another district municipality.

Interviewer: That’s interesting. Tell me more about that.

2: We see them in the local store, right. We bump into them every now and then.

3: The patients or the orthopedic surgeons?

2: The surgeons.

3: Yes, we do.

2: So….

1: Right. It would have been easier to refer patients to the south of Norway if I lived elsewhere.

(FG3)

This exchange indicates that the GPs would have felt less comfortable or committed to referring their patients to the local hospital if they had lived and worked in a community where they did not regularly encounter the local orthopedic surgeons. Although informal encounters are important, the GPs value formal communication from specialist healthcare services that introduces them to local hospital resources. For example, one GP appreciated a recent visit from the head of the orthopedic department:

Earlier this fall, the head of the orthopedic department visited us and explained, ‘Okay, currently, we have four orthopedic surgeons. All of them perform hip surgery, and two also perform knee surgery.’ Then we know. With this information, it’s easier to refer patients to them (FG1).

Such information not only helps GPs make referrals but also supports them in discussing healthcare options with their patients. One GP highlighted the importance of being well-informed:

Information, so we know what to tell our patients. It’s uncomfortable discussing [the local hospital], when I really don’t know what I’m talking about. What they do, how they are, how they operate. I don’t even know what they are doing in this town. So, more information would definitely help (FG5).

In summary, the GPs find that knowing specifics about the local services and being familiar with the orthopedic surgeons enhances their ability to communicate effectively and engage in decision-making processes with their patients, ultimately leading to more referrals to the local hospital.

#### Selection bias

While the GPs acknowledged and supported their patients’ right to choose their treatment provider, they noted limitations to this choice, emphasizing that hospitals ultimately determine which patients they accept for treatment:

1: I fully support the principle that everyone in this country should receive equal medical treatment. However, some hospitals tend to handle only straightforward cases, leaving the complications to the local hospitals.

2: I completely agree!

3: Typical [private hospital], right.

(FG2)

This discussion highlights one effect of ‘patient leakage’ where local hospitals disproportionately treat the sickest patients and those with complications, while other providers select less complicated cases. This pattern was also noted in another FG, where it was mentioned, “There is a selection bias because [hospital in southern Norway] doesn’t admit the sickest patients. If you have a severe heart condition, they will likely reject you” (FG4). Notably, the first quote suggests this selective admission is a privilege of private hospitals, but the second quote indicates that it also occurs in a public hospital in southern Norway.

The GPs’ perception of selection bias influenced their referral practices. Instead of actively involving patients in the decision-making process, they strategically guided them towards treatment providers they believed would admit them. One GP explained,

Patients with multimorbidity. That’s out of the question. Or perhaps not out of the question, but not all hospitals are willing to treat such complex cases. We must direct these patients to hospitals that are willing to help them. Otherwise, applying for treatment there is just a waste of time due to the inevitable rejections (FG4).

Consequently, instead of challenging the selection bias, the GPs inadvertently contributed to the disproportionate distribution of severe cases. This likely stemmed from their concern over waiting times and delayed treatment for patients, as suggested by the phrase “a waste of time”.

#### Concerns for post-operative care

As demonstrated in the previous sub-themes, GPs’ and patients’ knowledge and assumptions about hospitals and specialists significantly influence their decision-making processes regarding arthroplasty referrals. However, the FG discussions also highlighted concerns about the treatment trajectories beyond hospitalization. The GPs reported concerns, both among themselves and their patients, about post-operative care and follow-up. One major concern for GPs involved post-operative complications:

You get a feeling that if a patient had surgery in another part of the country, and there are complications, the local hospital isn’t very eager to look at it because they were not involved from the start. So, I usually base my decision on the local hospital on that. I have experienced a patient ending up with an amputation, several years after having surgery at a [hospital in southern Norway]. […] I’m not saying there are more complications there than elsewhere, but if there is trouble after an operation, it is perhaps good not to be far away from the hospital that did the job (FG5).

This quote underscores the importance of proximity to the surgical facility in case of post-operative complications, influencing GPs’ referral decisions. Additionally, patients’ access to post-operative rehabilitation also plays a crucial role:

1: You need to consider travel distances, both before and after treatment. What kind of rehabilitation options do you have? Where are these services located in relation to your home? These are important considerations.

2: Right.

3: Yes, because there’s less post-operative care available nowadays. Previously, routine rehabilitation was provided in [municipality hosting specialist healthcare services], but now, that’s rarely the case. Most patients now see a local physiotherapist.

(FG3)

This discussion emphasizes that GPs need to consider both travel distances and the availability of post-operative care when referring patients for surgery. It shows that previously, the availability of rehabilitation services in a specific municipality motivated GPs to refer patients to a hospital in the same area. However, this incentive has diminished as patients, regardless of their surgery location, are now typically referred to local physiotherapists. Some GPs suggested that certain hospitals in the southern part of Norway offer longer hospital stays, thereby reducing the need for post-operative care after discharge:

It’s not much help here. There are physiotherapists, but, well… Obviously, a hospital like [hospital in southern Norway] keeps the patients for 7–10 days – a practice they’ve been criticized for, because they have an overuse of rehabilitation services… But patients are not discharged until they have a certain level of functioning (FG1).

Given the limited availability of local rehabilitation services, patients and GPs may be motivated to choose a hospital further away if they are assured that patients are not discharged until they have sufficiently recovered. The lack of formal rehabilitation services also often forces patients to depend more on informal care from family members. In northern Norway, where many residents have family in the south, this familial connection influences their decision to travel there for surgery. One participant noted:

Patients often prefer to go to Oslo because they have family there, and that’s a significant factor when they are discharged from the hospital and need more support than what home care services can provide. Coping isn’t easy. Having a daughter in Bergen or Oslo affects their choice. This is a change from before (FG1).

This suggests that the absence of formal rehabilitation services and the dependence on family caregivers might contribute to ‘patient leakage’ from municipalities in Northern Norway to hospitals in the southern parts of the country.

### Societal and structural factors affecting referral practices

While the previous themes focused on interpersonal and local service-level factors, other sections of the FG discussions revealed that broader societal and structural factors also shaped the GPs’ referral practices.

#### Geographic and logistic factors

All municipalities involved in this study are situated in a region known for its vast distances, sparse population, and a trend of migration towards the southern parts of the country. These characteristics were echoed in the FG discussions. As highlighted in the sub-theme about post-operative care, GPs noted that proximity to family often influence patients to seek treatment in southern Norway. One GP explained, “I think it largely relates to demographics. There’s significant out-migration from Northern Norway. Consequently, it seems everyone has a daughter in South-Eastern Norway. So, it works out well for them” (FG2). Some GPs also noted that patients might choose hospitals in the south as it allows them to combine medical trips with personal travel. One GP remarked:

From what I’ve seen, many patients prefer traveling south rather than to [the two largest hospitals in Northern Norway]. I’m not entirely sure why. Some have mentioned that it’s easier to travel there. Others want to combine their visit with a weekend getaway (FG5).

Despite these personal preferences, a more pressing issue discussed was the region’s challenging travel logistics. The vast distances and limited transportation options make local travel both difficult and time-consuming. This was illustrated in the following exchange:

1: We’ve seen patients travel to various places in Northern Norway for orthopedic surgery. However, navigating the region can be quite challenging. It’s simpler to head to [hospital in south-eastern Norway].

2: Even getting to [hospital in Northern Norway] from here is a hassle.

1: Yes. Almost impossible.

(FG3)

The wide geographical spread and limited travel infrastructure hinder patients’ ability to choose a local treatment provider within the region. The distances make care travel both time-consuming and exhausting. As one GP put it, “Even though the travel time to [local hospital] is relatively short, it still requires a 1.5-hour drive. Psychologically, I think patients perceive it as just as convenient to fly elsewhere. Practically, there isn’t much difference” (FG4).

This discussion indicates that it is complicated for patients in this region to exercise their right to choose treatment provider to opt for a hospital within the region, both because distances are long and because travel options are few. The long geographical distances make traveling by car time-consuming and strenuous. Moreover, for many patients, air travel is the only viable option even within the region. The FG discussions revealed that it is often more convenient for patients to travel from the north to the south than to navigate between hospitals in the north:

1: It’s ironic that there are no direct flights between [two cities in same county] where the two hospitals are located.

2: Really!

1: But there aren’t any. And traveling via [third city in same county] takes six hours!

2: Mmmm….

1: So, it ends up being about three times longer to reach [neighbor hospital] than to fly to Oslo.

(FG1)

The FG discussions clearly demonstrated that the logistical challenges faced by patients in Northern Norway influence their hospital choice and access to healthcare. However, the discussions also revealed that these travel concerns do not necessarily influence the GPs’ preferences:

1: My observation is that when patients exercise their free hospital choice, they seldom choose locations within Northern Norway.

2: Yes.

3: It doesn’t really impact me….

2: [interrupts] Everyone here is accustomed to the distances.

3: …but I think it influences the patients. They opt for locations that are nearest to an airport.

4: I agree.

(FG2)

As such, within a landscape of shared decision-making and Free hospital choice, geographic and logistic challenges might still shape the GPs’ referral practices.

#### Local hospital disputes

The municipalities involved in this study are located in a region characterized by numerous and longstanding local hospital disputes. These conflicts revolve around the allocation of tasks and responsibilities among existing hospitals, possible closure of hospitals, as well as decisions regarding the locations of new hospitals. The FG discussions revealed that such disputes influence both the GPs referral practices and the patients’ preferences.

The GPs described their efforts to support their local hospital. One GP mentioned, “I’m actively promoting [local hospital] because I believe it’s a good facility. To keep it running, we need to direct patients there” (FG1). Through such statements, GPs recognized that their referral practices have broader societal impacts. However, they typically portrayed local political disputes over hospital locations and functions as issues affecting others more than themselves. For instance, they observed that GPs in other areas seemed more influenced by these disputes, “We remain loyal to our local hospital, but I’ve noticed that in other municipalities, people often prefer the university hospital” (FG1). They also noted that their patients tended to be more loyal to the local hospital compared to those from other areas: “Most of my patients prefer to be treated at our local hospital. However, I suspect patients from the other side of the mountain would rather go anywhere but here. It’s politics and propaganda” (FG3).

The GPs also discussed how local hospital patriotism and reluctance to seek treatment at competing hospitals were reinforced by social and traditional media. One GP explained, “It’s an echo chamber fueled by local media and Facebook. Conversations are amplified out of proportion, often intertwined with concerns about local employment” (FG4). Additionally, GPs shared insights into how local activist groups and media contribute to mistrust towards neighboring hospitals:

1: There’s a local group here vehemently advocating for a hospital here. It’s horrible.

2: And the local newspaper doesn’t help either. It constantly discredits the nearest hospital.

3: The paper has a dedicated hospital editor.

4: Exactly!

3: Published three times a week, each issue features something about the hospital, rarely anything positive.

2: Never positive.

1: There’s always some horror story, such as ‘Almost died from fruitless trip’.

(FG2)

The distrust and local patriotism significantly affect GPs referral practices. One GP noted, “The ongoing disputes have made many locals adamant about not using the hospital in the neighboring municipality. Since that’s where orthopedic surgeries are performed, I’m forced to refer them elsewhere” (FG5). If patients refuse treatment from nearby competitors, GPs must refer them to more distant hospitals, potentially leading to ‘patient leakage’ out of the region. Despite these challenges, GPs attempt to address such aversions during patient consultations. One recounted, “People stir each other up, agreeing not to support the neighboring hospital. I had a patient refuse to set foot there without any specific reason. After discussing his concerns, considering his age and the inconvenience of longer travel, he eventually agreed to the referral” (FG5).

## Discussion

Our findings reveal that the decision-making processes of Norwegian GPs regarding referrals for hip and knee arthroplasty surgeries are shaped by various intersecting factors. GPs engage in shared decision-making with their patients to varying degrees, primarily focusing on ensuring patients receive treatment with less emphasis on the choice of specific hospital. The GPs in our study presented themselves as supporters of local hospitals, while patients were the ones exercising their right to choose a different hospital under the ‘free hospital choice’ policy. Patients’ choices are increasingly informed and driven by specific healthcare demands. Additionally, GPs’ referral practices are influenced by service-level factors, including hospital reputation, GPs’ relationships with specialists, selection bias, and concerns about post-operative care accessibility. Broader societal and structural factors, such as geographic and logistical considerations, and local hospital disputes, also shape these practices. Consequently, our findings challenge the traditional view of shared decision-making as merely a collaborative process involving healthcare professionals and patients [6], suggesting that it may be too limited a definition in contexts involving ‘free hospital choice’.

This study add nuance to previous research that suggests patients typically rely on their GPs’ recommendations, assuming GPs are more informed about treatment options [5, 7–9]. The GPs in our study described their patients as well-informed and assertive about health matters and their rights, yet also influenced by myths, rumors, and local political agendas. Interestingly, several exchanges in the FGs indicated that GPs often choose not to challenge patients’ preferences by using benchmark information available from national clinical quality registries. This concurs with previous research [10]. Notably, the GPs preferred to remain “neutral”. Based on our data, we cannot determine whether GPs’ pursuit for neutrality stems from a belief that correcting patients’ misconceptions is beyond their mandate, or from other factors such as time constraints or uncertainty about how to obtain benchmark information. If true, the collaborative aspect of decision-making could be diminished due to GPs’ hesitance to use benchmark data. This issue warrants further investigation. Moreover, GPs’ reluctance to question patients’ choices might reflect a commitment to the principle of ‘free hospital choice’, prompting questions about whether this policy undermines the collaborative nature of decision-making processes.

The policy of ‘free hospital choice’ is presumed to improve quality and efficiency by fostering competition among health care providers [3, 4]. Concurrently, the hospital volume-outcome relationship is often used to justify centralizing specialized surgeries in fewer, larger facilities [22]. However, our research in a region with longstanding and ongoing local hospital disputes suggests that centralizing orthopedic surgery in fewer locations within each hospital enterprise may lead to increased ‘patient leakage’ out of the region. This occurs as some patients ‘vote with their feet’ and opt to travel to hospitals in southern Norway rather than “set foot in” the nearest facility offering orthopedic surgery, particularly if they perceive this hospital as undermining their local hospital interests. This phenomenon, elsewhere referred to as hospital bypassing [23], merits further investigation. In the Norwegian specialist healthcare system, where hospital funding is partially activity-based, ‘patient leakage’ from north to south results in reduced funding for hospitals in the northern part of the country. This funding decrease could ultimately compromise the principle of universal healthcare, which aims to provide equal access to healthcare services regardless of a person’s place of residence. Additionally, the GPs in our study noted a selection bias, indicating that not all patients experience a truly ‘free’ hospital choice. This is particularly concerning for patients with complex health issues, such as older patients and patients with multiple health conditions, who may not be eligible for treatment in the south, further exacerbating healthcare equity issues. A systematic review revealed that most patients are willing to travel further to reduce surgical risks. In contrast, older age and fewer years of formal education are linked to a higher tolerance for risk at local hospitals [24]. Another systematic review including countries where patients have the option to choose their healthcare provider, reported a negative association between patient mobility and factors such as advanced age or lower socioeconomic backgrounds [4].

A key finding from this study is the significant role of GPs’ familiarity and contact with specialists. GPs reported that their knowledge of local surgeons, coupled with their unfamiliarity with alternative hospitals and their surgical staff, inclined them to recommend the nearest hospital to their patients. This finding is consistent with a systematic review that indicated that referring physicians’ previous interactions with specialists influence their referral practices [8]. Both the continuity of local surgical staff and informal interactions between GPs and surgeons seem to foster loyalty to local hospitals. This issue should be considered by Norwegian healthcare authorities. A current strategy to maintain local hospital surgeries across Norway, including northern regions, involves hiring surgeons on short-term contracts from other areas or countries. While this approach is necessary, it might lead to ‘patient leakage’ if GPs, unfamiliar with these temporary surgeons, feel less inclined or obligated to recommend local hospitals. Therefore, more systematic and frequent contact between GPs and hospitals, such as through rotations and common professional forums, could enhance loyalty to the local hospital and thereby prevent ‘patient leakage’. Hospitals in the northern health region are likely more reliant on short-term locums to address staff shortage compared to hospitals in other health regions. However, we do not have official data regarding orthopedic surgeons to support this assumption.

Another key finding in this study was the impact of post-operative concerns. Several GPs noted that the lack of local rehabilitation services might contribute to ‘patient leakage’ to hospitals in southern Norway. This is exacerbated by the general trend of out-migration from the north to the south, resulting in many patients having family members in the south who can provide help and support during the post-operative phase. Therefore, strengthening local post-operative care and rehabilitation services could be considered a strategy to prevent ‘patient leakage’ from the north to the south. This implies that strategies to prevent ‘patient leakage’ should focus not only on GPs and surgical hospital units but also on other aspects of the patient’s journey, including access to rehabilitation services and post-operative care and support.

Our findings suggest that GPs referral practices and patients’ exercise of ‘free hospital choice’ in northern Norway are influenced by factors beyond healthcare services, including geographic and logistical constraints. These challenges highlight that preventing ‘patient leakage’ should not be seen solely as a healthcare issue but rather requires a cross-sectoral approach. Given that travelling between local hospitals in northern Norway remains more complex and time-consuming than travelling from the north to the south, many patients will continue to choose treatment in hospitals located in the southern regions of the country. A scoping review concluded that travel time might be a more significant factor than distance when evaluating healthcare access decisions in rural and remote communities [25].

This study was conducted within a specific and limited geographical area, which likely influenced our results. Additionally, it is important to consider the structure and funding of the Norwegian healthcare system – characterized by universal coverage and primarily public funding – when interpreting our findings. The composition of the FGs likely influenced the dynamics of the groups, as the familiarity among participants varied across the different FGs. A strength of this study is the deliberate selection of municipalities, which varied in population size and levels of ‘patient leakage’, to include in the analysis. Furthermore, the study benefited from data triangulation, achieved through five FGs with a total of 28 GPs. This allowed for space triangulation (collecting data on the same phenomenon across multiple sites) and person triangulation (collecting data from different groups of people) ([26]: 590). Additionally, the diverse methodological, disciplinary, and clinical backgrounds of the authors facilitated researcher triangulation, reducing the risk of biased interpretations ([26]: 592f).

## Conclusion

Our results suggest that shared decision-making is a complex process influenced by more than just the patient’s healthcare needs and preferences and the GP’s professional and clinical judgements. This study demonstrates that the decision-making processes affecting the choice of treatment provider for patients needing hip and knee arthroplasty are shaped by various factors at the patient, service, and societal levels. GPs acknowledge their vital role as gatekeepers in the referral process and as supporters of local hospitals. However, ‘patient leakage’ is affected by factors beyond their control. Therefore, policies aimed at preventing and reducing ‘patient leakage’ should not be viewed solely as a healthcare issue but should involve cross-sectoral measures.

## Supplementary Information

Supplementary Material 1.

## Data Availability

The dataset analyzed during the current study is available from the corresponding author on reasonable request.

## Abbreviations

FG: Focus groups
GP: General practitioners
