# Supplementary material for: Factors affecting general practitioners’ referrals of patients to hip and knee arthroplasty: a focus group study from Northern Norway
Source: BMC Health Serv Res. 2025 May 20;25:727. doi: 10.1186/s12913-025-12774-x (PMC12093783; doi:10.1186/s12913-025-12774-x)
Supplement: Supplementary file 1 — Supplementary Material 1. [file 12913_2025_12774_MOESM1_ESM.docx]

# Focus group interview guide: General practitioners

**Focus group frames**

- Provide information about the study and the focus group’s theme.
- Explain the use of the discussion, noting that results will be reported anonymously but organized by municipality.
- Address any uncertainties.
- Discuss the recording of the conversation and obtain consent.
- Inform about the duration of the focus group discussion and encourage active participation and the sharing of both positive and negative experiences.

**Introduction round**

- Municipality of residence and workplace.
- Educational institution.
- Work experience: Duration of work in the municipality. Experience from specialist healthcare?

**Referral practices**

- What influences your referral practices for patients being evaluated for or undergoing prosthetic surgery?
- Could you describe the decision-making process regarding where to refer the patient for treatment?
  - What do you emphasize?
- Where do you seek information and advice? How do you utilize information from national quality indicators and registries in your referral decisions?
- What are your considerations regarding travel distances and costs? How do such concerns influence decision-making?
- Have there been any changes in referral practices over the past five years? What might be the reasons for these changes?
- Which hospitals are your usual choices for referring patients? Do you have preferred treatment facilities? If so, why?
- If someone close to you needed these types of surgeries, what would your advice be?

**Patient involvement**

- Describe your approach to understanding a patient’s grasp of their illness and treatment.
- How do you perceive patient involvement in selecting the treatment location?
- Can you discuss how patients’ preferences emerge during consultations?
- What do you consider important for patients when choosing a treatment facility?
- How do you facilitate shared decision-making?
- Can you describe instances where it seemed inappropriate to inform the patient about their freedom to choose hospital?

**Relationship with hospital**

- How would you describe your collaboration with the local hospital?
- How is the collaboration concerning orthopedic patients locally?
- What makes a hospital attractive as a treatment location? What are your thoughts on the local hospital in this regard?
- How could the collaboration between the municipality and the local hospital be enhanced?

**Societal conditions**

- How do societal conditions influence patients’ choices and your referral practices?
- Hospital locations are being debated in several regions. Do these discussions affect the choice of treatment location?

**Review**

- Summarize the conversation and confirm if the participants have been understood correctly.
- Is there anything that should be emphasized?
- Is there anything that should be added?
- How was your experience participating in the focus group discussion?
